# Multilayer Alginate–Polycaprolactone Electrospun Membranes as Skin Wound Patches with Drug Delivery Abilities

**DOI:** 10.1021/acsami.0c07352

**Published:** 2020-06-23

**Authors:** Andrea Dodero, Marina Alloisio, Maila Castellano, Silvia Vicini

**Affiliations:** Department of Chemistry and Industrial Chemistry (DCCI), University of Genoa, Via Dodecaneso 31, 16146 Genova, Italy

**Keywords:** sodium alginate, polycaprolactone, electrospinning, multilayer membranes, wound healing patches

## Abstract

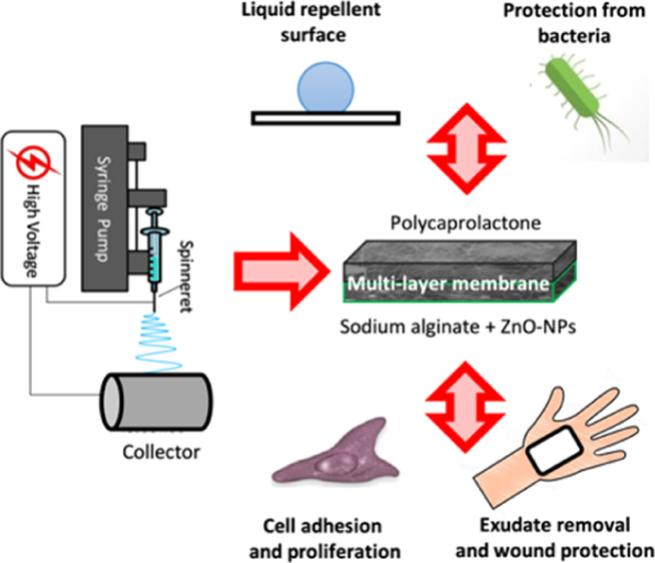

A multilayer nanofibrous membrane consisting of a layer of polycaprolactone and one of physically cross-linked alginate-embedding ZnO nanoparticles is prepared via electrospinning technique as potential wound healing patches with drug delivery capabilities. A washing–cross-linking protocol is developed to obtain stable materials at the same time removing poly(ethylene oxide), which was used here as a cospinning agent for alginate, without interfering with the membrane’s peculiar nanofibrous structure. The mechanical behavior of the samples is assessed via a uniaxial tensile test showing appropriate resistance and manageability together with a good thermal stability as proved via thermogravimetric analysis. The polycaprolactone external layer enriches the samples with good liquid-repellent properties, whereas the alginate layer is able to promote tissue regeneration owing to its capability to promote cell viability and allow exudate removal and gas exchanges. Moreover, using methylene blue and methyl orange as model molecules, promising drug delivery abilities are observed for the mats. Indeed, depending on the nature and on the dye-loading concentration, the release kinetic can be easily tuned to obtain a slow controlled or a fast burst release. Consequently, the proposed alginate–polycaprolactone membrane represents a promising class of innovative, simple, and cost-effective wound healing patches appropriate for large-scale production.

## 1 Introduction

Nowadays, skin chronic diseases (e.g., diabetic ulcers, psoriasis, and so on) and traumatic damages (e.g., burns, stab accidents, surgical events, and so on) are a great healthcare issue as they usually require long, expensive, and not always satisfactory medical treatments. Until now, autologous skin grafting represents the clinical “gold standard” in wound treatments, but its use is highly limited by the dimension and the thickness of the damaged area. Consequently, allogeneic or xenogeneic skin grafting is commonly employed. Nevertheless, it has a high risk of immune rejection and disease transmission.^1−3^ Taking into account all these disadvantages, wound healing patches, which are capable of promoting tissue regeneration simultaneously offering a protection from the external environment, have gained increasing interest in the last decade, representing an extremely attractive solution to overcome the limitations of traditional therapies. Such products are required to be completely harmless for the patient, highly effective from the clinical point of view, and mechanically appropriate for handling and application.^4^ In this regard, the electrospinning technique has emerged as an easy and affordable approach to prepare nanofibrous scaffolds with an architecture morphologically similar to the fibrillar constituents of the native extracellular matrix (ECM). The high superficial area of the nanofibers and their porous organization are indeed able to significantly improve cell viability, at the same time avoiding the wound getting in contact with bacteria and providing structural integrity and stability owing to their great tensile strength.^5,6^ In addition, steam cells, drugs, grown factors, and nanoparticles can be easily incorporated within the mat structure and used to further benefit the tissue regeneration.^7^ In recent years, the use of biocompatible and biodegradable natural polymers, such as polysaccharides [e.g., chitosan, collagen, sodium alginate (SA), and so on], in combination with the electrospinning technique has been widely investigated in order to obtain more and more efficient wound patches.^8−11^ However, besides their good biological response, polysaccharide-based electrospun scaffolds are often difficult to obtain owing to the poor processability of the raw materials^12^ and consequently require the use of a cospinning agent (e.g., synthetic biopolymers) and a cross-linker able to stabilize the nanofibers, which could however lead to a considerable decrease of the material biocompatibility.^13,14^ Moreover, such membranes often lack the appropriate mechanical and water-related properties to confer good manageability and protection from the external environment, thus hampering their efficient use in large-scale wound treatments.

SA is a linear polysaccharide mainly extracted from the cell wall of brown algae and widely employed for food, technological, and biomedical applications.^15−19^ One of the main reasons for alginate success is its ability to undergo a physical cross-linking reaction when in contact with bivalent ions, which avoids the need of highly toxic chemical cross-linkers such as glutaraldehyde and epichlorohydrin.^20^ In previous works,^21,22^ the preparation of an alginate-based electrospun membrane embedding ZnO nanoparticles (ZnO-NPs) was optimized and the development of an ionic cross-linking protocol coupled with a specific washing procedure able to completely remove the cospinning agent [i.e., poly(ethylene oxide) (PEO)] was reported. The obtained mat was characterized by a very promising biological response in terms of cell adhesion and proliferation, which were in fact comparable to those of a collagen-based commercial membrane (see Figure S1 for the detailed results), as well as by strong antibacterial and antibacteriostatic properties conferred by the nanoparticle presence (see Table S1 for the detailed results), a good mechanical resistance, and appropriate water-related properties.^22^ Besides the aforementioned promising findings, the tendency of alginate to adsorb a great amount of water and the difficulty to obtain a sufficient sample thickness prevent a good wound protection provided by the membrane itself.

In the present work, in order to avoid such undesirable effects, for the first time, a multilayer electrospun membrane consisting of an external layer of polycaprolactone (PCL) and an internal layer of physically cross-linked SA embedding ZnO-NPs was prepared. PCL was especially selected because of its biodegradability, solubility in not toxic organic solvents, ease of processability via electrospinning, and marked hydrophobic properties.^23,24^ The obtained multilayer membrane was characterized by thin and homogeneous nanofibers on both its sides, a highly porous structure, suitable mechanical properties, and good manageability. The external PCL layer showed a marked hydrophobic nature, thus providing efficient liquid-repellent and protection abilities toward the environment, whereas the internal alginate layer was able to promote tissue regeneration by removing the wound exudates, preventing bacteria proliferation, allowing gas exchanges, and providing the ideal environment to the cells. Moreover, the multilayer membrane was proved to represent a potential drug delivery system (DDS)^25^ with tunable release kinetics depending on both the nature of the incorporated drug and its loading concentration, thus opening the way to applications in treatments of both chronic and traumatic skin diseases.

## 2 Experimental Section

### 2.1 Materials

SA of medium viscosity with a viscosity-average molecular mass *M̅*_v_ = 350 kg mol^–1^ and a M/G ratio of 0.5^12^ was obtained from FMC Biopolymers. PCL with a number-average molecular mass *M̅*_n_ = 80 kg mol^–1^ was purchased from Solvay Chemicals. PEO with *M̅*_v_ = 900 kg mol^–1^, Triton X-100, zinc acetate dihydrate (ZnAc·2H_2_O), sodium hydroxide (NaOH), strontium chloride hexahydrate (SrCl_2_·6H_2_O), sodium chloride (NaCl), monosodium phosphate (NaH_2_PO_4_), disodium phosphate (Na_2_HPO_4_), methylene blue (MB), methyl orange (MO), glacial acetic acid (GAA), acetone (Ac), and absolute ethanol (EtOH) were obtained from Sigma-Aldrich.

### 2.2 Methods

#### 2.2.1 Solution Preparation and Rheological Characterization

ZnO-NPs and an SA-based solution were prepared according to our previous works.^21,22^ Briefly, ZnO-NPs were synthetized via a “green” approach using alginate itself as a stabilizing agent by keeping a mixture of 1 mL of NaOH 1 mol L^–1^, 2 mL of ZnAc 0.25 mol L^–1^, and 5 mL of SA 1% wt solution at 80 °C for 30 min. SA powder was then solubilized in a water suspension containing 0.25% wt of ZnO-NPs at room temperature under slow stirring to achieve its complete solubilization. PEO powder was subsequently added and the system was kept in agitation until homogeneity was reached. Finally, Triton X-100 was added in a concentration of 1% wt, maintaining the final solution under stirring for 24 h. The total polymer concentration was 3.5% wt with a SA/PEO ratio equal to 70/30. PCL solutions were prepared with dissolving for 2 h at *T* = 50 °C and under magnetic stirring the polymer powder with a concentration of 10, 20, or 30% w/v in GAA, Ac, or GAA/Ac mixtures in a volumetric ratio of 1/1 or 3/1. Then, solutions were kept under stirring at room temperature for 22 h prior to the electrospinning process to ensure that an equilibrium state was reached.

Steady-state viscosity measurements were carried out on PCL solutions by a rotational rheometer Physica MCR 301 (Anton Paar, GmbH Austria) equipped with a Peltier heating system and a solvent trap kit. The experiments were carried out at *T* = 20 ± 0.2 °C in the shear rate range 1–1000 s^–1^ using a double-gap geometry (DG26.7). To evaluate the PCL degradation because of possible hydrolysis phenomena, viscosity measurements were carried out on a sample system (i.e., 30% w/v GAA/Ac 3/1 mixture) at different times after the solution preparation (i.e., *t* = 0 corresponds to when the solution was removed from the heater).

#### 2.2.2 Electrospinning and Washing/Cross-Linking Procedure

A Doxa Microfluidics Professional Electrospinning Machine equipped with a drum collector was used to prepare the multilayer membrane. In a typical experiment, 20 mL of PCL solution was first electrospun using as processing parameters a spinneret-collector distance of 20 cm, a voltage of 15 kV, an infuse rate of 1 mL h^–1^, a needle inner diameter of 0.4 mm, and a drum rotation speed of 100 rpm. The total time of this step was of 20 h. Subsequently, to obtain the multilayer sample, 20 mL of the SA-based solution was electrospun directly on the PCL membrane using a spinneret-collector distance of 15 cm, a voltage of 12.5 kV, an infuse rate of 0.75 mL h^–1^, a needle inner diameter of 0.4 mm, and a drum rotation speed of 100 rpm. The total time of this step was of 26 h. The obtained mat had a size of 600 × 400 mm (length × width), whereas its average thickness was estimated by using a byko-test 4500 Fe/NFe (BYK Gardner, Germany). Once peeled off from the collector, the mat was immersed in a 3% w/v SrCl_2_ aqueous solution for 4 h in order to induce the alginate cross-linking and at the same time to remove the cospinning agent, rapidly washed with EtOH, and dried under vacuum at *T* = 50 °C for 24 h before being characterized.

#### 2.2.3 Morphological Investigation

Both sides of the electrospun membrane were analyzed by scanning electron microscopy (SEM) using a Hitachi TM3000 benchtop SEM microscope operating at a 15 kV acceleration voltage. A good conductivity of the samples was achieved with a thin layer of silver sputter-coated using a Quorum Q150R ES.

#### 2.2.4 Fourier-Transform Infrared Spectroscopy

Fourier-transform infrared spectroscopy (FTIR) was carried out on the polymer powders and on both sides of the multilayer mat. The spectra were collected in the wavelength range 400–4000 cm^–1^ with a 4 cm^–1^ resolution using a Bruker Vertex 70 instrument operating in attenuated total reflection mode.

#### 2.2.5 Thermogravimetric Analysis

Thermogravimetric analysis (TGA) was used to evaluate the thermal degradation profile of the polymer powders as well as of the multilayer membrane. A Mettler-Toledo TGA/DSC1 STARe System was employed in dynamic mode from 30 up to 700 °C with a heating rate of 10 °C min^–1^ under a continuous nitrogen flow of 80 mL min^–1^.

#### 2.2.6 Mechanical and Water-Related Investigation

The mechanical behavior of as-prepared and cross-linked multilayer samples was assessed on rectangular specimens (40 × 10 × 0.2 mm) via uniaxial tensile tests by using a displacement-controlled dynamometer Instron 5565. Sample testing was carried out at room temperature with an elongation rate of 25 mm min^–1^. A preload of 0.1 MPa was applied to ensure the correct sample loading. The Young’s modulus (*E*), the tensile strength (σ_r_), and the elongation at break (ε_r_) were evaluated via software from the stress–elongation curves. Measurements were repeated 5 times for each sample.

The water contact angle (WCA) of both PCL and cross-linked SA sides of the multilayer membrane was measured by an Attension Theta Lite optical tensiometer and used to evaluate the ability of the sample to interact with water. The moisture content (MC) of the membrane was assessed after a treatment at *T* = 110 °C under vacuum for 24 h according to eq 1
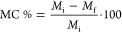
1where *M*_i_ and *M*_f_ are the initial and the final weights of the sample, respectively. The water vapor permeability (WVP) of the multilayer membrane was evaluated following the ASTM E96-95 standard test method by using eq 2(26)
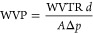
2where WVPR is the water vapor transmission rate, *d* is the sample thickness, *A* is the area of the sample able to permit the vapor diffusion, and Δ*p* is the partial vapor pressure difference between the two sides of the sample.

#### 2.2.7 Uptake–Release Capability

UV–vis spectroscopy measurements were carried out to investigate the adsorption and desorption properties of the multilayer membrane using a UV-1800 spectrophotometer (Shimadzu, Japan). Quartz cuvettes with an optical length of 1 cm were used for the experiments. MB (positive charged) and MO (negative charged) dye colorants were employed as drug model molecules. The adsorption peaks were assigned by literature at 663 nm for MB and at 464 nm for MO.^27^ Colorant solutions were prepared in deionized water with a dye concentration of 5, 10, 20, 40, 80, or 160 mg L^–1^ and employed as simulated drug-loading media.

To investigate the uptake kinetic, small portions of the mat were dipped in 3 mL of the 5, 10, or 20 mg L^–1^ loading solutions and kept under continuous stirring at room temperature. UV–vis spectra of each solution were collected every 30 min for a time period of 6 h and the adsorption capability was then calculated as follows (eq 3)
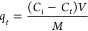
3where *C*_i_ and *C*_*t*_ are the initial colorant concentration and the colorant concentration at time *t*, respectively, *V* is the volume of the loading solution, and *M* is the mass of the sample; *C* values were calculated by the UV–vis spectrum adsorption intensity by using a calibration line.

The multilayer membrane portions were then rapidly washed with deionized water to remove the superficial not-adsorbed colorant molecules and immersed in 3 mL of phosphate buffer saline (PBS) to investigate the desorption kinetic. The pH of the release medium was 7.4 and the temperature 37 °C in order to simulate the physiological conditions. UV–vis spectra of the release solutions were measured every 30 min for a time period of 6 h and the percentage cumulative release (eq 4) was calculated as
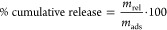
4where *m*_rel_ is the mass of colorant released from the sample and *m*_ads_ is the mass of the colorant initially loaded within the sample.

The isotherms of adsorption of the cross-linked multilayer membrane were assessed in the whole concentration range of the loading solutions. Small portions of the sample were immersed in 20 mL of the media for 7 days at room temperature. The equilibrium adsorption capacity *q*_e_ and the equilibrium colorant concentration *C*_e_ were then calculated as reported above.

## 3 Results and Discussion

### 3.1 PCL Solution Rheological Properties

**Figure 1:**
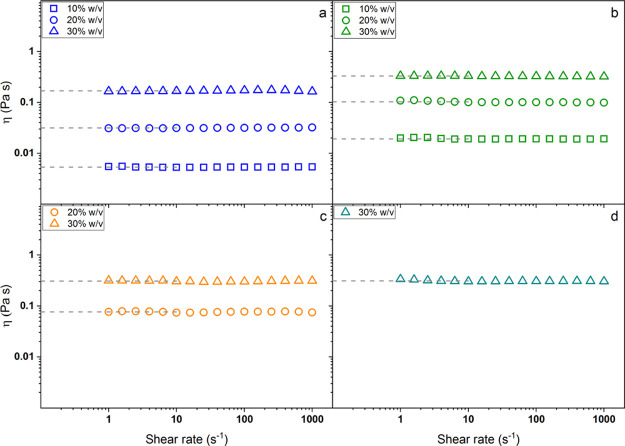
Steady-state viscosity curve of PCL solutions in Ac (a), in GAA (b), in GAA/Ac 1/1 mixture (c), and in GAA/Ac 3/1 mixture (d). Dashed lines represent the fitting of the experimental data with a straight line at low shear rate values.

Solution viscosity represents one of the main factors affecting the electrospinning process and the consequent morphology of the nanofibers. A too low viscous solution usually leads to inhomogeneous fibers characterized by the presence of a high number of bead-like structures, whereas a too high viscosity is undesirable as it causes negative effects on the system processability. Moreover, the high evaporation rate of organic solvents can help the deposition of the fibers but often induces the formation of a highly viscous semisolid at the end of the spinneret, leading to a not continuous electrospinning process. Therefore, the choice of both solvent type and polymer concentration represents a key aspect in the electrospinning process. Figure 1 reports the flow behavior of PCL solutions with different concentrations in Ac, in GAA, and in two mixtures of these solvents measured 24 h after their preparation. The use of a double-gap geometry and a solvent trap kit was fundamental here in order to limit as much as possible the solvent evaporation and obtain reliable results.

In the investigated shear rate range, all solutions were characterized by an almost constant viscosity value, thus showing with good approximation a Newtonian behavior. Such a finding is ascribable to the low molecular mass of PCL (*M̅*_n_ = 80 kg mol^–1^), which, owing to the shortness of the polymer chains, prevents the formation of a “dense” polymer network with a high number of entanglements, reducing the viscoelastic response of the system. To better compare the solution rheological properties, the zero-shear viscosity η_0_ (viscosity when the shear rate tends to zero) was calculated by fitting the experimental data at low shear rate values with a straight line according to the Newtonian model.^28,29^

The obtained η_0_ values are summarized in Table 1.

**Table 1:** Zero-Shear Viscosity Values for the Tested Solutions 24 h after Their Preparation

sample	η_0_·10^2^ (Pa·s)
10% w/v_Ac	0.54
20% w/v_Ac	3.2
30% w/v_Ac	16.9
10% w/v_GAA	1.9
20% w/v_GAA	10.3
30% w/v_GAA	32.9
20% w/v_GAA/Ac 1/1	7.6
30% w/v_GAA/Ac 1/1	30.8
30% w/v_GAA/Ac 3/1	31.1

Independently of the solvent type, a higher polymer concentration corresponded to a higher solution viscosity because of the higher number of chains in the solution.^30−33^ However, the solvent highly influenced the viscosity values. Indeed, PCL solutions in GAA were characterized by a significantly higher viscosity than those in Ac most likely because of a different affinity between the polymer–solvent couple. PCL is in fact reported to have a higher affinity for acetic acid with respect to Ac according to Hansen solubility parameters,^34^ which in turn leads to a positive contribution to the chains’ hydrodynamic volume (i.e., the polymer chains assume a more expanded random-coil conformation), with a consequent increase of the solution viscosity.^35^ Interestingly, PCL solutions in GAA/Ac mixtures showed viscosity values very similar to the solutions in pure GAA independently of the solvent ratio, thus indicating that acetic acid has a predominant effect with respect to Ac in influencing PCL chain conformation. Taking into account the obtained η_0_ values and the data of other works reported in literature,^36,37^ only the solutions with a polymer concentration of 30% w/v were selected to be electrospun. However, as PCL is easily subjected to hydrolysis phenomena in acidic conditions, as widely reported in the literature,^38,39^ the viscosity of the 30% w/v GAA/Ac mixture was evaluated for a time period of 48 h after the solution preparation (i.e., *t* = 0 corresponds to when the solution was removed from the heater and allowed to cool down at room temperature). Figure S2 reports the flow sweep curved obtained at different times with the correspondent *h*_0_ values, which were calculated as abovementioned, reported in Table S2. Besides a considerable decrement of the viscosity that could be observed with respect to the initial system and corresponding to a molecular mass reduction, the process seemed to stop after a time period of 6 h and significative differences could not be detected after 24 or 48 h. Thus, it is rather safe to assume that in the investigated acid environment, PCL solutions reached an equilibrium state 6 h after their preparation and could be stored or electrospun for a couple of days without the occurrence of viscosity-related effects.

### 3.2 Morphological Aspects

First, the electrospinning process of PCL in pure solvents was investigated. PCL in pure Ac could not be continuously electrospun because of the occurrence of an intense clogging phenomenon (i.e., formation of a semisolid deposit at the tip of the needle) affecting the process stability, as similarly reported by other research groups.^40,41^ On the contrary, PCL in pure GAA led to a continuous polymer jet without any difficulties. However, the obtained membrane was highly sticky and not mechanically resistant, with only small pieces that could be peeled off by the collector. Such a result is probably ascribable to the high boiling point of GAA, which hinders the complete solvent evaporation, therefore contributing to an inhomogeneous membrane structure and a consequent poor mechanical consistency. Otherwise, the electrospinning of PCL in both the tested GAA/Ac mixtures was stable and led to a consistent membrane with a good manageability, despite a slight clogging effect being detected for the GAA/Ac 1/1 system. Consequently, the PCL 30%_GAA/Ac 3/1 solution was selected as the most appropriate to prepare the external side of the investigated multilayer mat. Then, the SA-based solution containing ZnO-NPs was directly electrospun on the PCL membrane as optimized in our previous works.^21,22^ A thickness of 150 ± 15 μm was measured for the PCL layer, whereas the SA-based one showed a thickness of 50 ± 10 μm (total thickness was measured as 200 ± 15 μm). The obtained composite sample was peeled off the collector and subjected to a washing–cross-linking protocol optimized in order to remove the cospinning agent (i.e., PEO), simultaneously avoiding the alginate solubilization in aqueous media without modifying the mat’s nanofibrous structure. In particular, the immersion of the sample for 4 h in a water solution containing Sr^2+^ ions led to the stable cross-linking of alginate according to the “egg-box” model.^16,42^ On the contrary, PCL was not affected by such a treatment, being completely water-insoluble, whereas PEO was removed from the membrane (see the following section for the detailed results), owing to its high solubility in aqueous environments.

**Figure 2:**
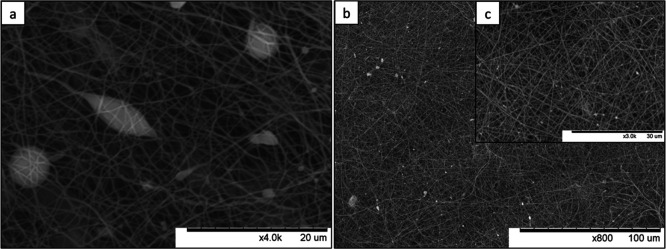
SEM images of the cross-linked membrane. PCL nanofibers are shown in (a), SA-based nanofibers in (b) at a low magnification and in (c) the inset at high magnification.

Figure 2 shows the morphology of the PCL layer (Figure 2a) and of the SA-based layer (Figure 2b). The morphology shown here refers to the final sample after the washing–cross-linking process.

The PCL layer was characterized by homogenous nanofibers with a mean diameter of 300 ± 50 nm uniformly distributed in the membrane structure with only a small number of detectable defects. Moreover, the pores between the fibers appeared to be highly regular with a dimension of a few micrometers, conferring to the sample a great porosity. The SA layer was instead characterized by much thinner nanofibers, whose diameter was around 100 ± 30 nm, and a homogeneous porous structure similar to that of the ECM. As thoroughly investigated in our previous studies,^21,22^ the synthetized ZnO-NPs were characterized by irregular structured clusters because of the interactions occurring between the single nanoparticles, whose size was about 20–30 nm as shown in Figure S3a. The almost complete absence of aggregates observed in Figure 2 and the energy-dispersive system results, which are reported in Figure S3b, proved the good dispersion of the nanoparticles within the alginate fibrous structure, thus demonstrating the proficiency of electrospinning as an easy and cheap approach to prepare nanocomposite materials.

### 3.3 Composition and Thermal Properties

**Figure 3:**
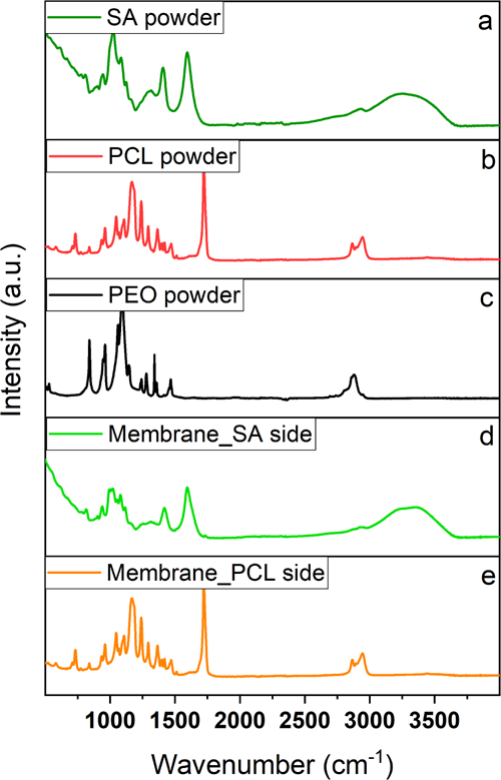
FTIR spectra for SA powder (a), PCL powder (b), PEO powder (c), SA side of the cross-linked membrane (d), and PCL side of the cross-linked membrane (e).

In order to evaluate the effective removal of the cospinning agent (i.e., PEO) from the alginate-based layer, FTIR was employed to detect the typical spectroscopic signals of each polymer by comparing them with those of the double-layer membrane after the washing–cross-linking process. Figure 3 reports the obtained results.

The main adsorption peaks of alginate (Figure 3a), PCL (Figure 3b), and PEO (Figure 3c) were assigned according to literature. For SA, the broad unstructured band at 3600–3000 cm^–1^ corresponded to the stretching vibrations (ν) of the hydroxyl groups; the asymmetric (ν_a_) and symmetric (ν_s_) stretching of the carboxylic groups were found at 1599 cm^–1^ and at 1417 cm^–1^, ν(C–O–C) were observed at 1085 cm^–1^ and at 1026 cm^–1^, and the signals at 940 and 904 cm^–1^ were ascribable to ν(C–O).^17,21^ For PCL, −CH_2_ stretching vibrations occurred at 2900 cm^–1^, ν(C=O) were observed at 1722 cm^–1^, bending modes (δ) of −CH_2_ groups were detected at 1472 cm^–1^, and ν(C–O) were detected at 1172 cm^–1^.^43^ For PEO, the main absorption peaks were observed at 2890 cm^–1^ because of the −CH_2_ stretching vibrations and at 1148, 1096, and 1078 cm^–1^ owing to the combination of ether group and methylene group stretching vibrations.^44^

By comparing the spectrum of pure alginate powder (Figure 3a) with that of the alginate-based membrane layer (Figure 3d), the same absorption peaks could be observed with some slight shifts occurring because of establishment of electrostatic interactions between the polymer chains and both ZnO/NPs and Sr^2+^ ions.^45^ Moreover, the typical signals of PEO were not observed, suggesting the removal of the co-pinning agent, thanks to the long immersion in the aqueous cross-linking solution. Finally, the PCL membrane layer (Figure 3e) was characterized by the same spectrum of the polymer powder (Figure 3b), indicating its ability to maintain the initial chemical structure despite the several applied treatments.

**Figure 4:**
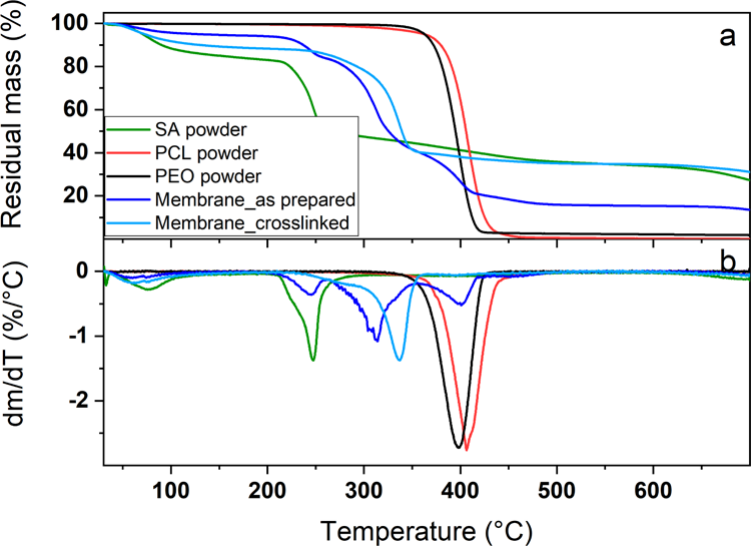
Thermal degradation profiles (a) and related first derivative curves (b) of SA, PCL, and PEO polymer powders together with those of the multilayer membrane before and after the washing–cross-linking process.

The thermal stability of the prepared mats was assessed via TGA with the degradation profiles reported in Figure 4a and the related first derivate curves (DTG) in Figure 4b.

The SA powder (green line) showed three different mass loss steps at *T* < 120 °C because of the residual humidity and physical bounded water, at *T* ∼ 250 °C corresponding to the degradation of alginate to form metal carbonates, and starting from *T* ∼ 650 °C owing to the complete burning of organic residues occurring at higher temperatures. On the contrary, both PCL (red line) and PEO (black line) powders were characterized by a single sharp degradation step occurring at *T* ∼ 400 °C. The as-prepared multilayer membrane (blue line) presented four different degradation peaks corresponding to the residual humidity vaporization (*T* < 120 °C), to alginate (*T* ∼ 250 °C), to PCL (*T* ∼ 310 °C), and PEO (*T* ∼ 400 °C) degradation. The lower temperature to which PCL thermal disruption occurred with respect to the polymer powder (*T* ∼ 310 °C instead of *T* ∼ 400 °C) is ascribable to the molecular mass reduction taking place during the solubilization in GAA and described in Section 3.1 (see Figure S1 and Table S1). Remarkably, after the washing–cross-linking treatment, only three degradation steps could be clearly detected for the multilayer sample (light blue line). The first one at *T* < 120 °C was again related to the residual humidity and bound water, the second one at *T* ∼ 275 °C was ascribable to alginate degradation, and the broad degradation step at *T* ∼ 340 °C could be attributed to PCL. Different effects can be clearly noticed here. First, the physical crosslinking of alginate can increase its thermal stability, thus shifting the degradation temperature at slightly higher values (*T* ∼ 275 °C instead of *T* ∼ 250 °C). Similarly, PCL degradation seems to be retarded compared to the as-prepared sample, which can be ascribable to an increase of the polymer molecular mass and/or crystallization degree occurring during the membrane drying step at *T* = 50 °C.^46^ Finally, the thermal peak corresponding to the degradation of PEO is not clearly present after the washing–cross-linking treatment, thus suggesting the complete removal of the cospinning agent and confirming the results obtained from FTIR.

In general, the thermal properties showed by the cross-linked multilayer membrane indicate good stability and a good resistance to the washing–cross-linking treatments to which it was subjected.

### 3.4 Mechanical and Water-Related Properties

The mechanical behavior and the water-related properties (i.e., WCA, MC, and water vapor permeability) are fundamental features to be considered in the development of wound healing patches. Indeed, such products must show mechanical properties similar to those of human skin^47−49^ being able to remove exudates, provide a sufficient gas exchange, and protect the wounds from the external environment.

**Figure 5:**
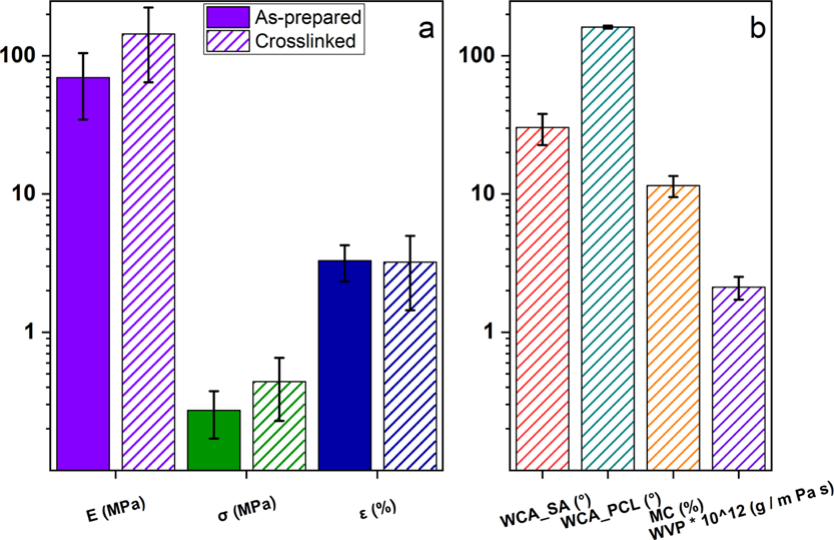
(a) Summary of the mechanical properties of the as-prepared (filled bars) and of the cross-linked membrane (dashed bars). (b) Summary of the water-related properties of the cross-linked membrane.

Figure 5a summarizes the mechanical properties (i.e., Young’s modulus *E*, tensile strength σ_r_, and elongation at break ε_r_) of the as-prepared (filled bars) and cross-linked (dashed bars) multilayer membrane. Stress-elongation curves are reported in Figure S4.

As clearly observed, the cross-linking process induced an increase of the sample mechanical stiffness with the Young’s modulus increasing from 70 to 145 MPa and the tensile strength from 0.27 to 0.44 MPa, whereas no significant changes were detected for the elongation at break that remained constant at a value around 3%. Indeed, the cross-linking of alginate forms physical bridges between the polymer chains, thus creating a stable polymer network that provides an improved mechanical response and stiffness. Besides the obtained values being in the suitable range to promote cell adhesion and to help skin and soft tissue regeneration,^50^ the prepared multilayer sample was characterized by lower mechanical properties compared to pure alginate electrospun mats, whose behavior was assessed and reported in our previous work.^22^ Such a phenomenon can be probably attributed to the PCL layer. Indeed, the mechanical properties of the PCL electrospun mats reported in literature clearly indicate a marked elastic behavior at the expense of the sample stiffness.^51,52^

Regarding the water affinity of the multilayer membrane, SA and PCL sides showed considerably different properties as summarized in Figure 5b. The alginate layer of the mat showed indeed a strong hydrophilic behavior with a WCA = 30°, in agreement with alginate ability to interact with water via hydrogen bonds and to adsorb great amounts of the aqueous solvent. Conversely, the PCL layer appeared to be highly hydrophobic with WCA = 160°, therefore demonstrating the polymer-repellent properties against aqueous solutions. Moreover, the low MC (∼10%) of the mat could undoubtedly help to preserve the sample properties over a long time period, whereas the high water vapor permeability (WVP) (2.2 × 10^–12^ g m^–1^ Pa^–1^ s^–1^) should provide a sufficient gas exchange with the external environment, thus promoting the cell viability. Notwithstanding, with respect to the monolayer alginate-based electrospun samples previously investigated,^22^ the membrane prepared here showed lower WVP values owing to both the hydrophobicity of PCL layer and its higher thickness.

Remarkably, the obtained results proved the possibility to use the prepared double-layer membrane as a promising and efficient way to protect skin wounds from the outside, at the same time providing an excellent environment to promote tissue regeneration.

### 3.5 Adsorption–Desorption Ability

Besides a nano- and microstructure resembling the ECM, an appropriate mechanical response, and good water-related properties, wound healing patches should also possess particular uptake and release abilities in order to include within their structure and subsequently deliver drugs, vitamins, and growth factors. Indeed, a slow controlled release of such molecule types can be helpful in the treatment of chronic skin damages (e.g., diabetes ulcers), whereas a fast burst release is important for traumatic episodes (e.g., stab wounds).

**Figure 6:**
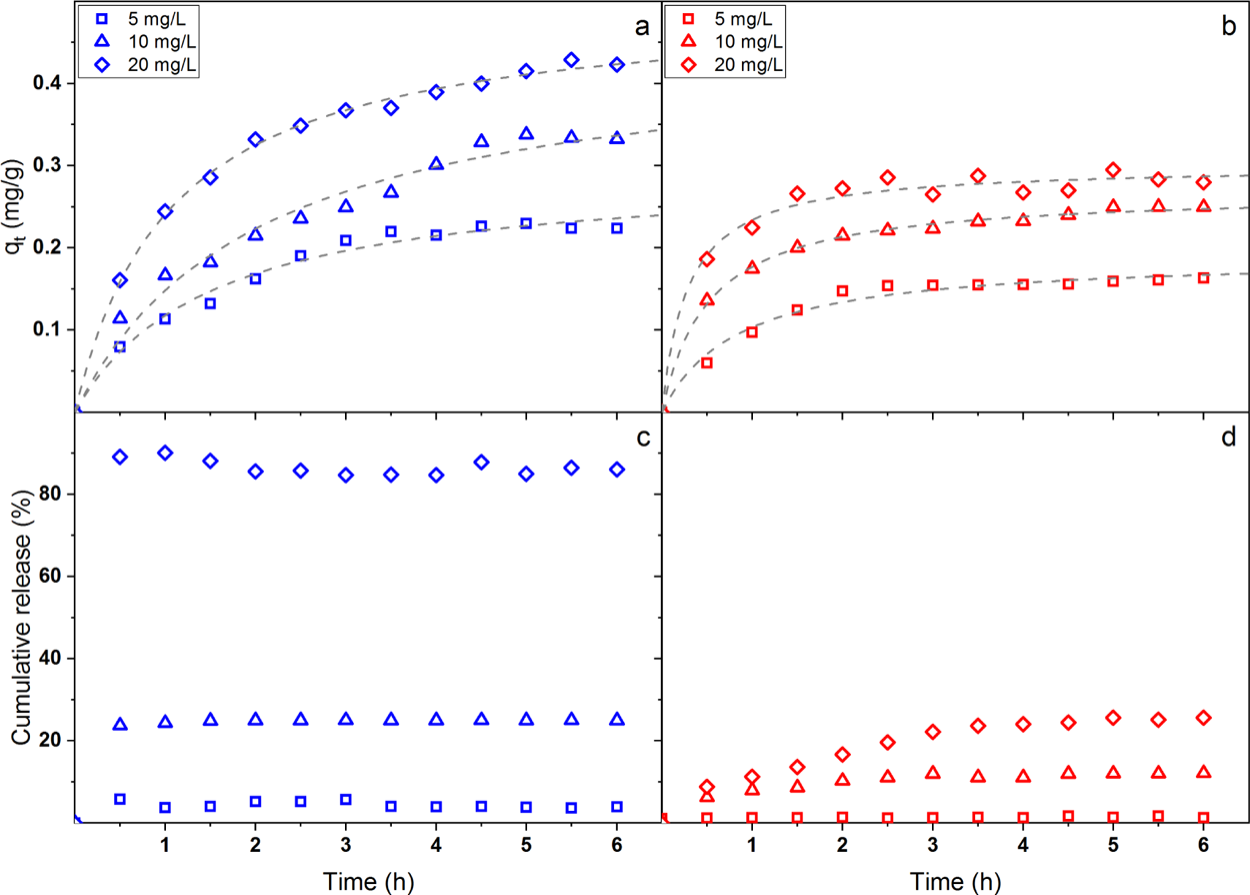
Adsorption kinetics of the multilayer membrane for (a) MB and (b) MO; dashed–dotted lines represent the fitting of the experimental data with the pseudo-second-order model. Release kinetics of the multilayer membrane for (c) MB and (d) MO.

As most of the molecules used in DDSs are positively or negatively charged, in the present work MB and MO colorants were used as cationic and anionic model drugs, respectively. The adsorption capacity *q*_*t*_ of the multilayer membrane in deionized water is reported in Figure 6a for MB and in Figure 6b for MO.

As expected, strong differences were observed in the adsorption capability of the membrane toward the two dyes. In particular, for MB, higher amounts were adsorbed, and the process continued even after 6 h of immersion in the loading solutions, especially for the most concentrated ones. On the contrary, for MO, besides lower *q*_*t*_ values, the adsorption phenomenon seemed to stop after only 3 h independently of the colorant concentration. The higher affinity observed for MB can be easily explained considering the negative charges present on the alginate backbone, which attractively interact with positive-charged molecules. However, as also MO was adsorbed into the membrane notwithstanding it should repulsively interact with alginate chains, also diffusion phenomena seemed to play an important role here and need to be considered. In this regard, the adsorption experimental data were fitted with two theoretical models according to a pseudo-first-order (eq 5) and a pseudo-second-order (eq 6) kinetic
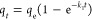
5
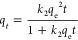
6where *q*_e_ is the equilibrium adsorption capability, *t* is the adsorption time, and *k*_1_ and *k*_2_ are the kinetic rate constants.^53^ For both dyes, the pseudo-second-order kinetic model was found to better fit the experimental data, therefore proving that the adsorption phenomenon occurs via both electrostatic and diffusion processes. More in detail, given the different obtained results, it can be supposed that for the prepared multilayer membrane, diffusive phenomena are preferred for negative-charged molecules, whereas electrostatic interactions dominate the adsorption of molecules with positive charges.^54,55^ In this regard, Figure S5 reports the difference in color between SA and PCL layers after the uptake of MB. As clearly observed, the SA layer appeared to adsorb much more dye with respect to the PCL layer, thus proving to some extent the predominance of electrostatic interactions in the described process. On the contrary, no significant color differences were observed after the adsorption of MO (data not shown). Such findings were further confirmed by the release kinetic occurring in PBS solution and are shown in Figure 6c for MB and in Figure 6d for MO, where a completely different behavior can be observed for the two colorants. MB was indeed immediately released independently of the loading concentration, whereas a slow and controlled release was observed for MO. Moreover, given the same loading concentration, the released amount of MB was considerably greater than for MO. The presence of ions in PBS helps to screen the charges of the alginate backbone, thus leading to the immediate release of MB, which is in fact mainly adsorbed via electrostatic interactions. On the contrary, as MO uptake is mostly governed by diffusion phenomena, the desorption process occurs slowly and only partially.^56^

**Figure 7:**
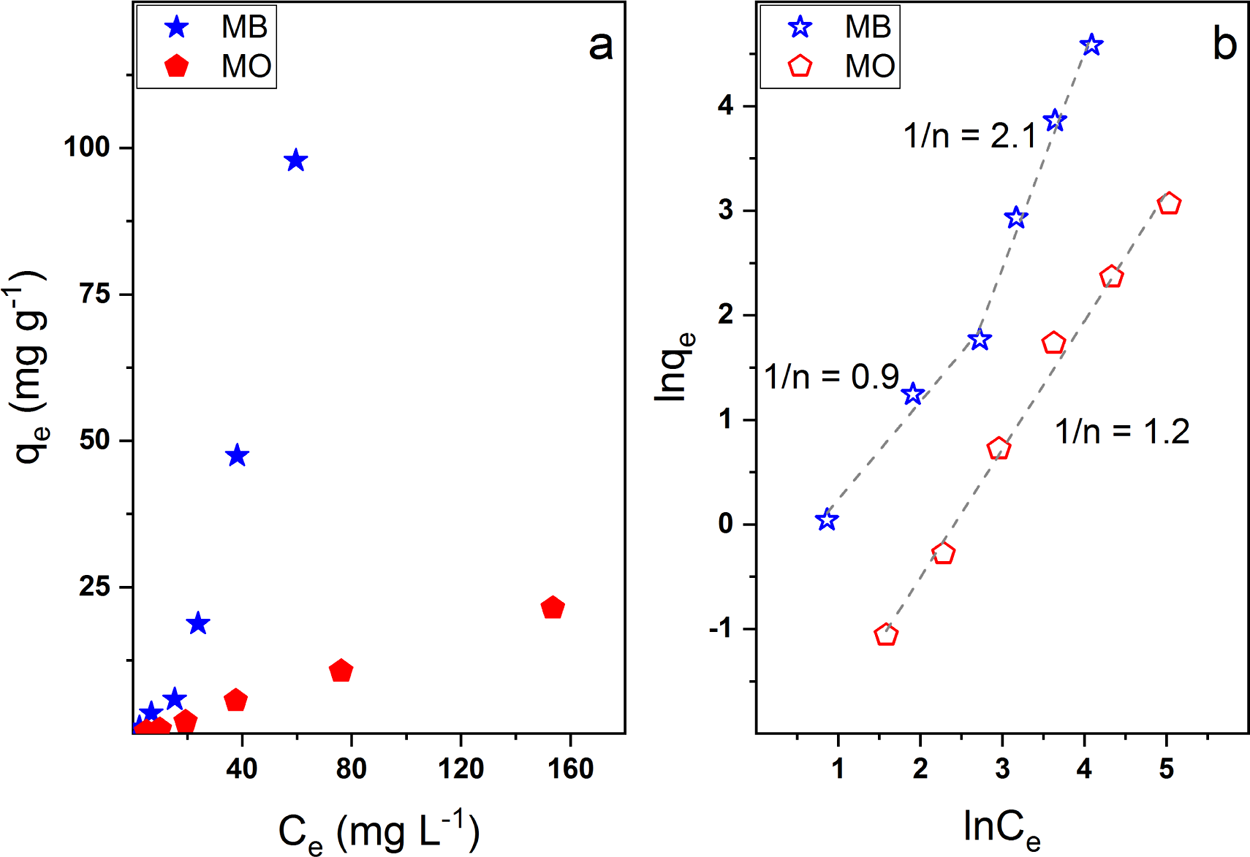
(a) Isotherms of adsorption of the multilayer membrane for MB (star symbols) and MO (rhombus symbols). (b) Linearized Freundlich model.

In both cases, the amount of colorant that was freed from the membrane seemed to be proportional to the loading concentration, which consequently assumes a key role in order to obtain DDS systems with appropriate release kinetics. In this regard, the isotherms of adsorption of the prepared multilayer membrane were investigated and the obtained results are shown in Figure 7a.

The equilibrium adsorption capability *q*_e_ was found to increase with the equilibrium concentration *C*_e_ for both colorants despite significantly higher values being obtained for MB mainly because of its greater affinity for alginate. In particular, a strong increment of *q*_e_ can be observed for MB when the loading concentration is raised above 20 mg L^–1^. Consequently, it can be rather safely supposed that MB uptake occurs at first mainly via electrostatic interactions but once all the available sites are “occupied”, for example, when the loading concentration is high enough, the diffusive phenomena assume a much important role and a fast increase of *q*_e_ is observed. On the contrary, a more regular behavior can be observed for MO as the electrostatic interactions do not significantly contribute to the adsorption process.

To describe in detail the adsorption isotherms, two different theoretical models were applied to the experimental data. The Langmuir model (eq 7) assumes that the equilibrium adsorption capacity is governed by the adsorption of a single adsorbate monolayer according to
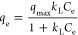
7where *q*_max_ is the amount of adsorbate required to form a complete monolayer and *k*_L_ is the Langmuir rate constant.^57^ The Freundlich model (eq 8) is instead based on the fact that the adsorption phenomenon occurs on a heterogenous surface as follows
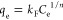
8where *n* is an empirical constant and *k*_F_ is the Freundlich rate constant.^58^

In both cases, the Langmuir model completely failed to describe the experimental values (fitting not shown), whereas a good agreement was found using the Freundlich model, whose linear fitting is shown in Figure 7b, thus indicating the coexistence of both electrostatic and diffusive adsorption phenomena. In particular, MO experimental data led to a single straight line able to describe the whole concentration range with a slope (i.e., 1/*n*) of 1.2. MB behavior was instead better described by using two different concentration ranges (i.e., 0–20 and 20–160 mg L^–1^) and slope values of 0.9 and 2.1 were obtained for the first and the second regions, respectively.

The parameter 1/*n* is related to the degree of curvature of the investigated isotherms and, in particular, 1/*n* > 1 is associated to S-type isotherms.^59,60^ Such behavior is usually observed for compounds containing polar groups which are in competition with water for the adsorption sites. More importantly, *n* values are indicative of the intensity of the process, which becomes more favored as *n* tends to 0. Here, as previously mentioned, the uptake of MB is governed by both electrostatic and diffusive phenomena, depending on the concentration, and seems to be highly favored above a certain point when the two assume a synergic nature, with *n* = 0.5.

The obtained results suggested that the prepared multilayer membrane represents an interesting and promising class of new products to be used as DDSs whose release kinetic can be easily modulated by selecting both the appropriate adsorbed molecule and the loading conditions.

## 4 Conclusions

Potential wound healing patches with drug delivery properties based on a multilayer alginate–PCL membrane embedding ZnO-NPs were for the first time prepared via electrospinning technique. A washing–physical cross-linking protocol, which consisted of the immersion for 4 h of the sample in an aqueous solution containing strontium ions, was especially developed in order to cross-link alginate, at the same time removing the used cospinning agent [i.e., PEO] without affecting the sample structure and biocompatibility. PCL external layer conferred to the membrane good mechanical properties and manageability, as well as strong liquid repellent abilities, thus representing an efficient protection from the external environment and helping the patch effective application. Additionally, alginate internal layer was able to considerably promote the cell viability, to remove exudates, and to allow gas exchanges, with ZnO-NPs enriching it with strong antibacterial and antibacteriostatic properties (as previously demonstrated^22^). Moreover, the prepared multilayer membrane was characterized by an important thermal stability (i.e., degradation temperature above *T* = 250 °C). Finally, promising drug delivery capabilities were observed and, above all, depending on the nature and concentration of the loaded molecules, different kinetic releases were obtained. Consequently, the prepared multilayer alginate–PCL mat could be efficiently used as wound healing patches for the treatment of both skin chronic disease and traumatic damages.

## Supporting Information Available

Assessment of the biological and antibacterial properties of alginate-based membranes embedding ZnO-NPs; evaluation of PCL solution viscosity decrease; ZnO morphological investigation; mechanical properties of the prepared multilayer membranes; and evaluation of the different MB uptake mechanisms for PCL- and SA-based layers (PDF)

## Notes

Data will be made available on request.
