# Supplementary material for: Multilayer Alginate–Polycaprolactone Electrospun Membranes as Skin Wound Patches with Drug Delivery Abilities
Source: ACS Appl Mater Interfaces. 2020 Jun 23;12(28):31162–71. doi: 10.1021/acsami.0c07352 (PMC8008386; doi:10.1021/acsami.0c07352)
Supplement: Supplementary file 1 — am0c07352_si_001.pdf [file am0c07352_si_001.pdf]

Supporting Information

Multi-layer alginate-polycaprolactone electrospun membranes as skin wound patches with drug delivery abilities

Andrea Dodero, Marina Alloisio, Maila Castellano*, Silvia Vicini*

Department of Chemistry and Industrial Chemistry (DCCI), University of Genoa, Via Dodecaneso 31, 16146, Genova, Italy

*corresponding authors:	maila.castellano@unige.it	(+39) 010 3538706
	ORCID: 0000-0002-0930-7522	
	andrea.dodero@edu.unige.it	(+39) 010 3538762
	ORCID: 0000-0002-1413-8508	

marina.alloisio@unige.it ORCID: 0000-0002-6155-2912

silvia.vicini@unige.it ORCID: 0000-0001-9369-1522

Content

S1. Assessment of the biological and anti-bacterial properties of alginate-based membranes embedding ZnO nanoparticles

S2. Evaluation of PCL solution viscosity decrease

S3. ZnO morphological investigation

S4. Mechanical properties of the prepared multilayer membranes

S5. Evaluation of the different MB uptake mechanism for PCL and SA-based layers

S1. Assessment of the biological and anti-bacterial properties of alginate-based membranes embedding ZnO nanoparticles

The biological response and the anti-bacterial properties of alginate/ZnO electrospun membranes were assessed as thoroughly described in reference 22. Here, Figure S1-a reports the results of the cytotoxicity test over a time period of 48h undoubtedly proving the safety of the proposed mats. The adhesion of mouse fibroblasts (L929) on the alginate-based mats is reported in Figure S1-b (gray bars) together with the cell proliferation (orange bars). Similarly, the results attained by using human keratinocytes (HaCaT) are shown in Figure S1-c (gray bars for the adhesion, purple bars for the proliferation). The data are compared with those obtained with a commercial collagen membrane as control. The consistency of the results demonstrated the capability of the proposed membranes to promote the cell viability, thus representing a promising class of wound healing products.

Additionally, the anti-bacterial properties of the mats were assessed against *E. Coli* bacteria as described in detail in reference 22. Table S1 summarizes the obtained results pointing out the strong effect of ZnO nanoparticles and confirming the possibility to use such membranes as infection control systems.

Table S1. Evaluation of bacterial adhesion on a collagen, alginate and alginate/ZnO-NPs membrane immersed for 5 h in PBS suspension of *E. coli* at 10^7 cells/mL and washed twice with sterile PBS.

Sample	*E. Coli* cell number
Collagen membrane	1180.18 ± 159.56
Alginate membrane	77.53 ± 5.74
Alginate/ZnO-NPs membrane	< 10

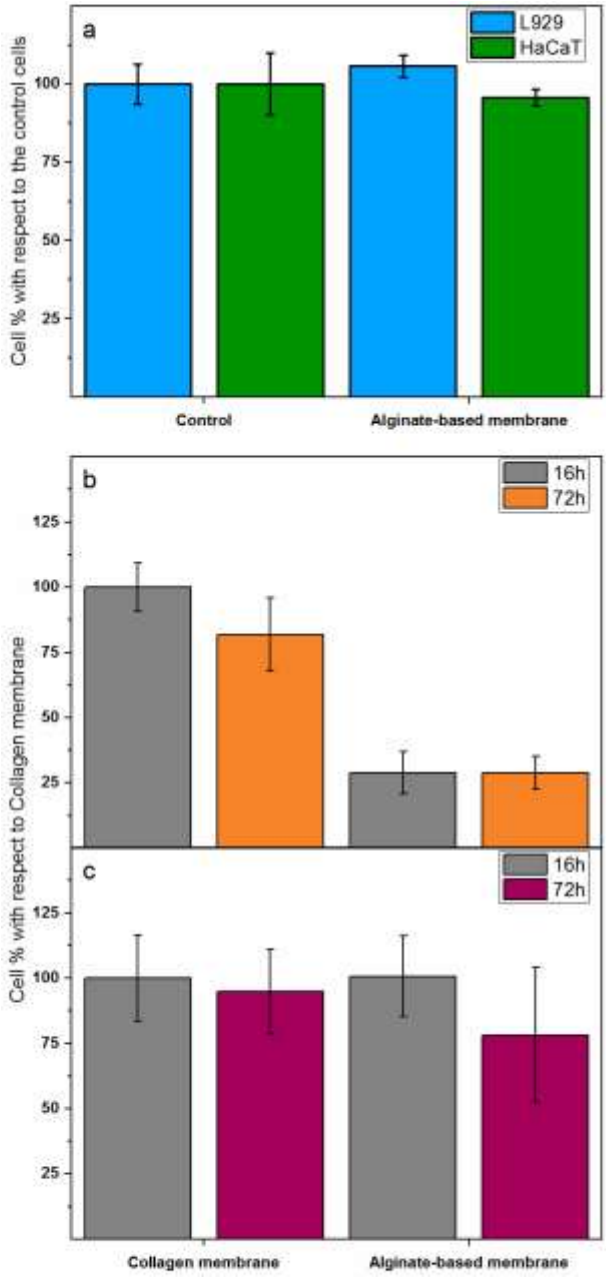

Figure S1. (a) Cell cytotoxicity by MTT test of alginate/ZnO-NPs membrane extracts obtained by soaking them in complete medium for 6 h and then added to L929 fibroblasts (blue bars) or HaCaT keratinocytes (green) bars) for 48 h. Cell adhesion and growth by MTT test of (b) L929 fibroblasts and (c) HaCaT keratinocytes on a commercial collagen and alginate/ZnO-NPs membranes after 16 h or 72 h of incubation. Results are expressed as cell percentages respect to the commercial collagen membrane.

S2. Evaluation of PCL solution viscosity decrease

Owing to the fact that PCL can easily undergo to hydrolysis phenomena, as broadly described in the literature, the viscosity of PCL 30% w/v glacial acetic acid/acetone solution was evaluated over a time period of 48h after its preparation. The experimental data were fitted with a straight line at low shear rate values in order to calculate the zero-shear viscosity (η_0). Figure S2 reports the obtained curved, whereas Table S2 summarizes the viscosity values depending on the time of investigation. Clearly, a strong hydrolysis phenomenon can be observed during the first 6h leading to a considerable decrement of the solution viscosity owing to the reduction of the macromolecular chain length and their capability to form entanglements. However, such process seems to slow down or even stop after 6h, with constant viscosity values observed after 24h and 48h.

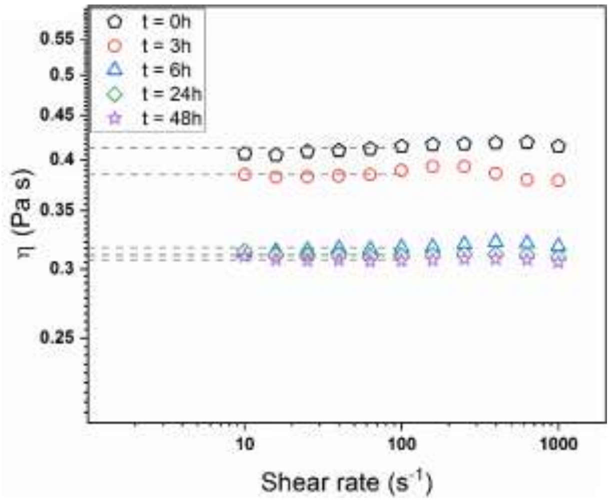

Figure S2. Flow behavior of the PCL 30% w/v GAA/Ac mixture at different time after its preparation.

Table S2. Zero-shear viscosity values of the PCL 30% w/v GAA/Ac mixture at different time after its preparation.

Time after preparation (h)	$\eta_0 \cdot 10^2$ (Pa·s)
0	41.3

3	38.5
6	31.7
24	31.1
48	30.7

S3. ZnO morphological investigation

The morphology of the synthetized ZnO nanoparticles is shown in Figure S3-a. ZnO-NPs were found to form irregular clusters with various dimensions, besides the single nanoparticles could still be recognised showing a dimension in the range 20-30 nm.

The distribution of ZnO nanoparticles within the electrospun alginate-based layer was evaluated via EDS. Figure S3-b indicates ZnO-NPs as red points homogenously distributed both on the surface and in the inner part of nanofibers. Green points are instead related to the presence of strontium, which was here used as crosslinking agents.

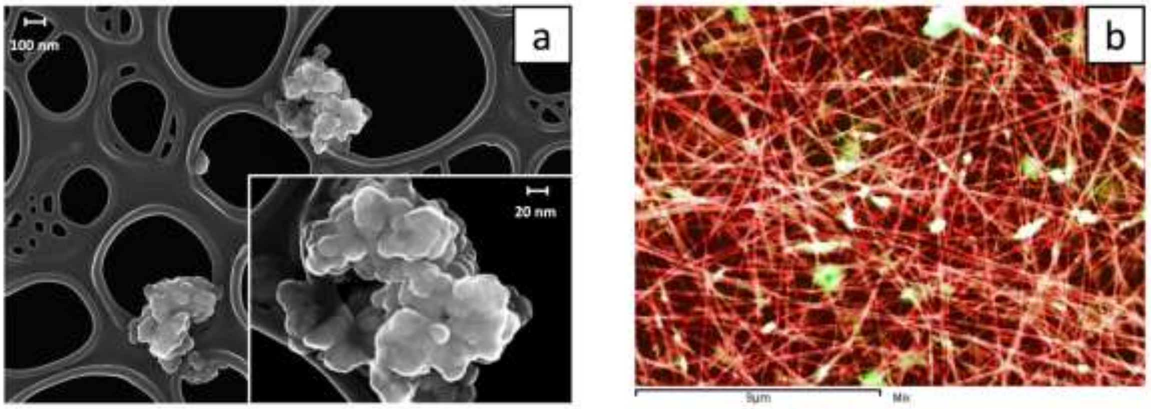

Figure S3. (a) SEM images of the synthetized ZnO nanoparticles at low and high (Figure inset) magnifications. (b) Distribution of ZnO nanoparticles (red points) within the alginate-based layer obtained via EDS.

S4. Mechanical properties of the prepared multilayer membranes

Figure S4 reports the stress-elongation curves obtained by the mechanical tensile tests performed on both the as-is (black squares) and crosslinked (red circles) multilayer membranes.

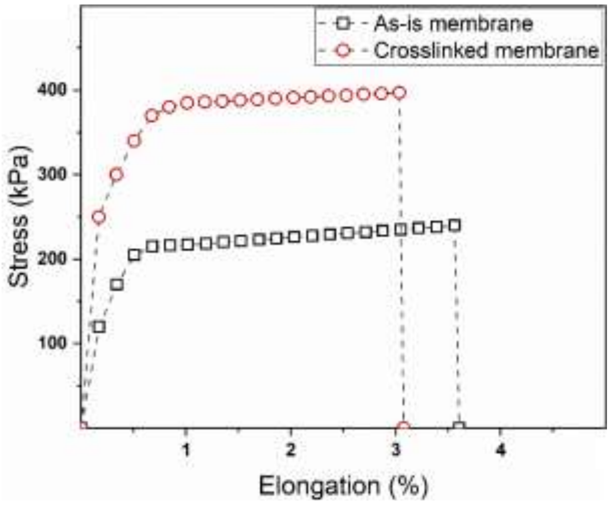

Figure S4. Stress-elongation curves obtained from the mechanical tensile test of the prepared multilayer membranes.

S5. Evaluation of the different MB uptake mechanism for PCL and SA-based layers

The color difference between PCL layer (on the left) and SA-based layer (on the right) of the multilayer membrane after the uptake of MB is shown in Figure S5. As clearly observed and as suggested by the fitting of the experimental data with several mathematical models, a much higher amount of dye was adsorbed by the alginate layer owing to the attractive electrostatic interactions occurring between them (i.e. MB is positive charged and SA is negative charged), besides diffusive phenomena could not be completely neglected.

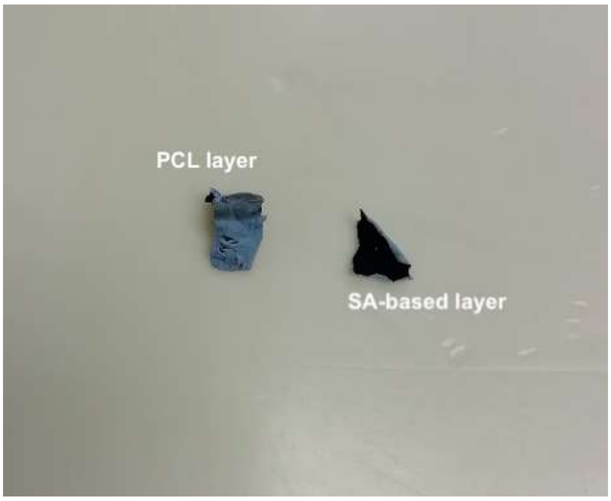

Figure S5. Colour of the multilayer membrane on both sides after the adsorption of MB.
